# Gut Microbiota Disorders Promote Inflammation and Aggravate Spinal Cord Injury Through the TLR4/MyD88 Signaling Pathway

**DOI:** 10.3389/fnut.2021.702659

**Published:** 2021-09-13

**Authors:** Zijie Rong, Yuliang Huang, Honghua Cai, Min Chen, Hao Wang, Guihua Liu, Zhiwen Zhang, Jiawen Wu

**Affiliations:** ^1^Department of Spine Surgery, Huizhou Municipal Central Hospital, Huizhou, China; ^2^Orthopaedic Institute, Huizhou Municipal Central Hospital, Huizhou, China; ^3^Department of Orthopaedics, Huizhou Municipal Central Hospital, Huizhou, China; ^4^Department of Spine Surgery, The People's Hospital of Longhua, Shenzhen Longhua Clinical Medical College of Guangdong Medical University, Shenzhen, China

**Keywords:** SCI, gut microbiota, TLR4, MyD88, GFAP, inflammation

## Abstract

**Background:** In spinal cord injury (SCI), systemic inflammation and the death of nerve cells in the spinal cord are life threatening. The connection between gut microbiota and signaling pathways has been a hot research topic in recent years. The Toll-like receptor 4/Myeloid differentiation factor 88 (TLR4/MyD88) signaling pathway is closely related to the inflammatory response. This study explored whether the gut microbiota imbalance could affect the TLR4/MyD88 signaling pathway to regulate SCI to provide a new basis for SCI research and treatment.

**Methods:** An SCI model was constructed to study the influence on the injury of gut microbiota. 16S amplicon sequencing was used to identify the diversity and abundance of gut microbes. Fecal microbiota transplantation was performed in mice with SCI. ELISA was used to detect the serum levels of pro-inflammatory and anti-inflammatory factors in mice. Hematoxylin and eosin staining was used to observe SCI in mice. Immunofluorescence was used to detect the rates of loss glial fibrillary acidic protein (GFAP), neuronal nuclear protein (NeuN), and ionized calcium-binding adapter molecule 1 (IBA1) in the spinal cord as indicators of apoptosis. The expression of the TLR4/MyD88 signaling pathway was detected by qRT-PCR and western blotting.

**Results:** Significant differences were observed in the gut microbiota of SCI mice and normal mice. The gut microbiota of SCI mice was imbalanced. The levels of pro-inflammatory cytokines tumor necrosis factor-α, interleukin (IL)-1β, and IL-6 in SCI mice were increased, as was the level of the toxic induced nitric oxide synthase. The levels of anti-inflammatory factors IL-4, transforming growth factor-β, and IL-10 were decreased, as was the level of arginase-1. The apoptosis rates of GFAP, NeuN, and IBA1 were increased. The TLR4/MyD88 signaling pathway was activated. In the SCI group, inflammation increased after fecal transplantation, apoptosis of GFAP, NeuN, and IBA1 increased, and SCI was more serious.

**Conclusion:** The TLR4/MyD88 signaling pathway promotes the death of nerve cells by inducing inflammation. Gut microbiota dysregulation can lead to aggravated SCI by activating the TLR4/MyD88 signaling pathway.

## Introduction

SCI has a significant socio-economic impact on society reflecting the considerable life-long health care expenditures (1). In SCI, synaptic connection loss, demyelination, and axonal injury destroy signal propagation, and neurons undergo mechanically induced cell death (2). Excessive inflammation may hinder nerve repair and regeneration. Many studies have been conducted to improve the treatment of SCI by reducing secondary inflammation (3). However, SCI treatment remains a medical concern worldwide.

Oral broad-spectrum antibiotics produce an imbalance in the gut microbiota due to the perturbation of the gut microbiota. This alteration in the gut microbiota can exacerbate neurological damage and spinal cord pathology after SCI. Dysbiosis develops when the composition of the gut microbiota is altered such that beneficial non-pathogenic gut bacteria (i.e., probionts) are depleted or become overwhelmed by pathogenic inflammatory bacteria (i.e., pathobionts). Autoimmune diseases (e.g., multiple sclerosis, type I diabetes, and rheumatoid arthritis), allergies, and metabolic disorders have been linked to gut dysbiosis (4–7). Recent data in humans and rodent models suggest that changes in the gut microbiota are disease-mitigating factors that could affect system physiology and pathophysiology. The exact mechanism remains unclear.

Changes in the composition of the gut microbiota and its metabolites will transfer from the intestine to the intestinal wall and cross the ruptured intestinal barrier, intensifying inflammation and affecting various organs (8). Fecal microbiota transplantation (FMT) protects Parkinson's disease model mice by inhibiting neuroinflammation and reducing Toll-like receptor 4/Tumor necrosis factor-alpha (TLR4/TNF-α) signaling (9). Studies show that SCI-induced gut dysbiosis is involved in the development of anxiety-like behavior following SCI, since both gut dysbiosis and anxiety-like behaviors were significantly reduced following treatment with an FMT. TLR4 is expressed on the cell membranes of microglia, the principal immune cells of the central nervous system (CNS). It was postulated that microglial activation participates in I/R injury through the release of growth factors, chemokines, regulatory cytokines, and other toxic mediators (10). Changes in the gut microbiota lead to neuroinflammation and intestinal damage through intestinal leakage and TLR4 activation (11). TLR4 promotes microglial apoptosis by activating the phosphoinositide 3-kinase (PI3K)/AKT pathway after SCI (12). Following SCI, necrotic astrocytes induce high inflammatory response genes encoding TLR4 and myeloid differentiation primary response gene 88 (MyD88) (13). Overexpression of the TLR4 receptor leads to enhanced astrocyte proliferation/microglial cell response and exacerbates SCI (14). However, it has not yet been reported that the gut microbiota exacerbates SCI through the TLR4/MyD88 signaling pathway. We used FMT to explore whether the gut microbiota of SCI mice could exacerbate SCI and systemic inflammation in mice.

TLR4 is activated by lipopolysaccharide (LPS), a component of the cell envelope of gram-negative bacteria. Activated TLR4 induces the production of pro-inflammatory mediators to destroy the bacteria (15). Dysregulation of the host response to LPS can lead to a systemic inflammation called sepsis (16). Typically, before TLR4 is activated, it binds to CD14 proteins anchored in cholesterol and sphingolipid-rich microdomain (termed a raft) in the plasma membrane (17). MyD88 is mainly responsible for directing intracellular signal transduction, which is essential for innate immune regulation (18). MyD88 is an anchoring adaptor protein that integrates and transduces intracellular signals generated by the TLR and interleukin (IL)-1 receptor (TLR/IL-1R) superfamily (19). We are interested in exploring whether activation of the TLR4/MyD88 signaling pathway could aggravate SCI and systemic inflammation in mice.

Although previous research results support that activation of the TLR4/MyD88 signaling pathway may trigger spinal cord cell inflammation and apoptosis, there has been no relevant research on whether the SCI gut microbiota could aggravate SCI through the TLR4/MyD88 signaling pathway.

## Materials and Methods

### Cell Culture

Mouse microglia BV2 cells and LPS (1 μg/mL) were used to establish like a microglial model of inflammation. The number of cells in each well was normalized to the average number of cells in the control condition (100%). Then cultured for 7 days. The Control group comprised BV2 cells and the LPS group comprised LPS-treated BV2 cells for 24 h.

### Animals

Thirty-two, 6-week-old C57BL/6 mice weighing 25 ± 2 g were purchased from Hunan Slack Jingda Experimental Animal Co., Ltd. The handling of animals during the experiment complied with the *Guiding Opinions on the Good Treatment of Laboratory Animals*, published by the Ministry of Science and Technology in 2006. 10 mice were selected as the Sham group, and the remaining 22 were used to construct the SCI model.

### SCI Model

After anesthetizing the mouse, laminectomy was performed to expose the spinal cord at T10. A spinal cord impactor (68,097, RWD, CA, USA) was used to create injuries by dropping a 5-g rod onto the spinal cord from a height of 6.5 cm. Immediately afterward, the overlying muscle was sutured and the skin were sutured. The animal's bladder was emptied three times a day until reflex control of bladder function was restored. The procedure in the Sham operation group was similar to that in the SCI group, except that no substantial injury was caused to the spinal cord. Twenty-two animals were used for modeling. Two died, representing a modeling success rate was 90.91%. On the 1st day after operation, hind limb paralysis and motor deficits appeared in mice. 20 successfully modeled animals were divided into four groups (*n* = 5): SCI (spinal cord injury mice), SCI+PBS (spinal cord injury mice have received the enema with PBS), SCI + Sham-FMT group (spinal cord injury transplanted with Sham mouse feces transplanted), and SCI + SCI-FMT group (spinal cord injury transplanted with SCI mouse feces). 10 mice of Sham were divided into two groups (*n* = 5): Sham (A laminectomy without SCI damage), Sham+PBS (Mice without SCI damage have received the enema with PBS). Seven days after operation, locomotor behavior was monitored Subsequently, mice were euthanized with an overdose of barbiturate (150 mg/kg) and spinal cord tissues at the injury epicenter were isolated for quantitative real-time PCR (qRT-PCR) and western blot.

### Analysis of rRNA Amplicons

After collecting fecal samples from normal and SCI mice, three qualified Control groups (10.1, 8.1, and 9.1) and seven SCI DNA samples from the SCI group (1.1, 2.1, 3.1, 3.2, 4.1, 4.2, and 4.3) were detected. The qualified library was sequenced using an Illumina pe150 device. The raw data were used for later information analysis. The representative sequences of each operational taxonomic unit (OUT) were annotated to obtain the corresponding species and species abundance distributions. At the same time, OTU abundance and alpha diversity were calculated to obtain species richness and evenness information in samples and common and unique OTU information among different samples or groups. Multi-sequence alignment of OTUs was performed and a phylogenetic tree was constructed. To further explore the differences in community structure among grouped samples, *t*-test, metastat, lefse, analysis of similarities, and multiple response permutation procedures were used to test the significance of species composition and community structure of grouped samples.

### ELISA

Concentrations of stimulating follicle hormone, progesterone, luteinizing hormone, and testosterone in serum samples were determined using an ELISA kit (CSB-E04634m, CSB-E08054m, CSB-E08326m, CSB-E04639m, CSB-E04594m, CSB-E04741m, CSB-E04726m, CusaBio, Wuhan, China) according to the manufacturer's instructions, and were repeated three times. The liquid was discarded and the wells dried without washing. Biotin-labeled antibody working solution (100 μL) was added to each well, covered with a new plate, and incubated at 37°C for 1 h. Horseradish peroxidase-labeled avidin solution (100 μL) was added to each well, covered with a new plate, and incubated at 37°C for 1 h. Substrate solution (90 μL) was added to each well to develop the color at 37°C in darkness for 15–30 min. Within 5 min after the termination of the reaction, the optical density of each well was measured using a microplate reader at 450 nm.

### Hematoxylin-Eosin Staining

Sections were heated at 60°C for 1–2 h. Each section was immersed in solutions of 100, 95, 85, and 75% ethanol for 5 min each. Hematoxylin was applied for 5–10 min, the section was washed with distilled water, and PBS back to blue. Eosin was applied for 3–5 min followed by rinsing with distilled water. Each section was dehydrated using a graded series of ethanol solutions gradient alcohol (95–100%) for 5 min each. The final solution was removed and replaced by xylene for 10 min. The sections on a slide were sealed with neutral gum and examined by microscopy. Each group of three mice were selected and a cross section was selected on each mouse.

### Quantitative Real-Time PCR

Total RNA from colon and spinal cord cells was extracted using TRIzol (15596026, Thermo Fisher Scientific, Waltham, MA, USA). The sample RNA was reverse transcribed to cDNA according to the instructions of the reverse transcription kit (cw2569, Kangwei Century Company, China). Subsequently, real-time PCR was performed on a fluorescence quantitative RCP instrument (QuantStudio1, Thermo, USA) using a UltraSYBR Mixture (CW2601, CWBIO, China). The reaction system is 20 μL. Fluorescence quantitative PCR was performed in a fluorescence quantitative RCP instrument (QuantStudio1, Thermo, USA). The reaction conditions were denaturation at 95°C for 10 min, denaturation at 94°C for 15 s, annealing at 60°C for 30 s, for 40 cycles. The primer internal reference was β-actin. The primer sequences are shown in Table 1. With 2 μg cDNA as template, the relative quantitative method (2^−ΔΔCt^ method) was used to calculate the relative transcription level of the target gene: ΔΔCt = Δ experimental group –Δ Control group, ΔCt = Ct (target gene)-Ct (β-actin). The experiment was repeated three times.

**Table 1:** Primer sequences.

**Gene**	**Sequences (5^**′**^-3^**′**^)**
TLR4	F: AGACACTTTATTCAGAGCCGTTG
	R: AAGGCGATACAATTCCACC
MyD88	F: TCCCCAAGAAAGTGAGTCTCC
	R: AAAGTACAAACACGAGCCCTT
IκBa	F: AGCATCTCCACTCCGTCCTG
	R: ACATCAGCACCCAAAGTCACC
p65	F: TAGCCAGCGAATCCAGACCAACA
	R: TGGGTCCCGCACTGTCACCT
β-actin	F: ACATCCGTAAAGACCTCTATGCC
	R: TACTCCTGCTTGCTGATCCAC

### Western Blot

Total protein was extracted from colon and spinal cord cells using the Ripa Kit (r0010, Solarbio, China). The protein concentration was determined using the BCA method. Quantitative analysis was performed in accordance with the different concentrations. Protein were resolved was by 10% SDS-PAGE and transferred to a nitrocellulose membrane by electroporation. The membrane was incubated with 5% skim milk for 2 h at room temperature to bind with nonspecific protein, and then incubated at 4°C. Primary antibodies, rabbit anti-TLR4 (1:500 dilution, ab13867, Abcam, Cambridge, UK), rabbit anti-MyD88 (1:1000, 23230-1-AP, Proteintech, Rockford, IL, USA), rabbit anti-p-IkBα (1:2000, ab133462, Abcam), rabbit anti-IκBα (1:2000, 10268-1-AP, Proteintech), rabbit anti-p-p65 (1:1000, #3033, Cell Signaling Technology, Danvers, MA, USA), rabbit anti-p65 (1:1000, ab32536, Abcam), followed by rinsing three times for 10 min each time using Tris-buffered saline-Tween. This was followed by exposure to horseradish peroxidase-conjugated goat anti-mouse IgG (1:5000, sa00001-1, Proteintech). The membrane was immersed in Supernal Plus (k-12045-d50, Advansta, USA) for luminescence development. β-actin was used as an internal reference. Protein bands were scanned using Scion image software.

### Immunofluorescence Double-Staining

The sections were deparaffinized with water. Sections were stained to detect apoptosis using a terminal deoxynucleotidyl transferase-mediated digoxigenin-dUTP nick end-labeling (TUNEL) assay. The sections were placed in three xylene solutions for 20 min each time. They were then treated with 100, 95, 85, and 75% ethanol for 5 min each. The sections were then soaked in distilled water for 5 min and then placed in citrate buffer solution (pH 6.0) and boiled by continuous microwaving for 23 min, and cooled to room temperature. Each section was then placed in sodium borohydride solution at room temperature for 30 min and rinsed with water for 5 min. This was followed by exposure to Sudan black dye solution at room temperature for 5 min and rinsing with water for 3 min. Following addition of normal serum (10%) and bovine serum albumin (5%) for 60 min, each section was exposed to terminal deoxynucleotidyl transferase (TDT) buffer, 34 uL deionized distilled water, 10 μL 5× equilibration buffer, 5 μL fluorescein isothiocyanate-12-Dutp Labeling Mix, and 1 uL recombinant TDT. The primary antibodies incubated overnight at 4°C were anti-GFAP (1:100, 16825-1-AP, Proteintech), anti-IBA1 (1:100, 10904-1-AP, Proteintech), and anti-NeuN (1:100, AB177487, Abcam). The sections were rinsed with PBS three times, 5 min each time, and then treated with 50 to 100 uL of anti-rabbit, rabbit, and rabbit-IgG-labeled fluorescent antibody at 37 °C for 90 min. Following rinsing with PBS three times for 5 min each time, cell nuclei were stained by 4', 6-diamidino-2-phenylindole (DAPI) at 37 °C for 10 min. Each section was rinsed three times with PBS for 5 min each 3 time, sealed with buffered glycerin, and examined by fluorescence microscopy. The confocal images of cells were sequentially acquired with Zeiss AIM software on a Zeiss LSM 510 confocal microscope system. Each group of three mice was selected and a cross section was selected on each mouse.

### Statistical Analyses

All data were analyzed using GraphPad Prism 8.0 software (GraphPad Software, La Jolla, CA, USA). The results were expressed as mean ± standard deviation (SD). Unpaired *t*-test was used to compare the two groups with a normal distribution. Comparisons among multiple groups were conducted using one-way analysis of variance (ANOVA), followed by Tukey's *post hoc* test. Differences were considered statistically significant at *P* < 0.05.

## Results

### LPS Induces Inflammation in BV2 Cells and Promotes Apoptosis

**Figure 1:**
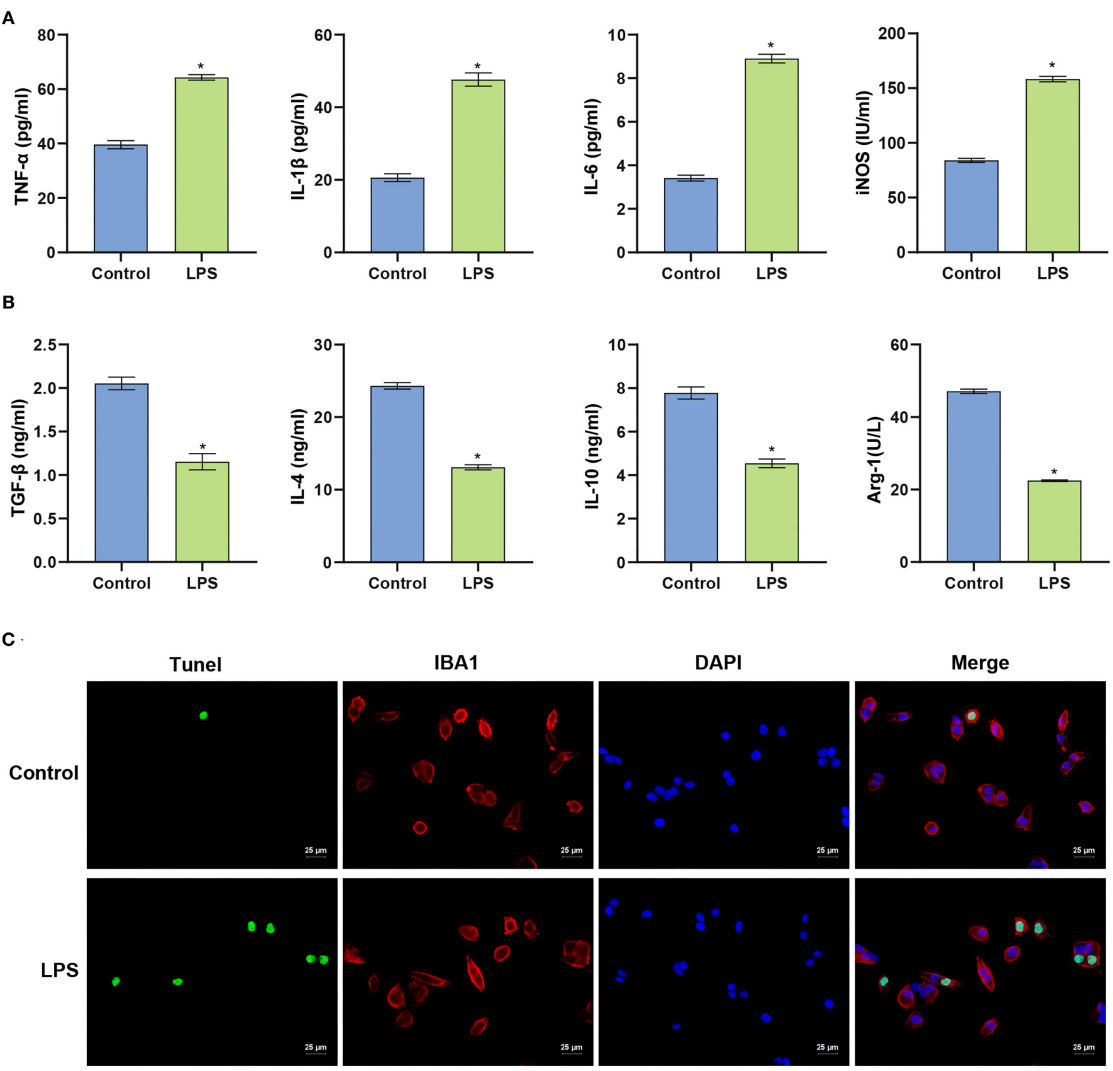
LPS induces apoptosis of BV2 cells. **(A)** Level of the measured pro-inflammatory factors in BV2 cells. **(B)** ELISA determination of the levels of anti-inflammatory factors in BV2 cells. **(C)** TUNEL assay (Scale bar = 25 μm). The unpaired *t*-test was used to analyze comparisons between two groups. ^*^P < 0.05 compared with the control group.

We constructed an *in vitro* microglial model of inflammation in mice using BV2 cells to evaluate the inflammatory response and survival of BV2 cells. ELISA was used to identify the levels of pro-inflammatory and anti-inflammatory factors released by BV2 cells. Compared with the Control group, LPS-treated cells released more pro-inflammatory factors (IL-1β, TNF-α, and IL-6), induced nitric oxide synthase (iNOS) was significantly increased (*p* < 0.001). Anti-inflammatory factors (TGF-β, IL-4, IL-10) and arginase 1 (Arg-1) were significantly decreased (*p* < 0.001). These data indicate that the LPS-induced inflammatory response in BV2 cells was exacerbated (Figures 1A,B). Compared with the Control group, the fluorescence intensity of TUNEL increased in the LPS group. Apoptosis in the spinal cord of mice was examined in more detail (*p* < 0.001) (Figure 1C). The collective findings indicate the LPS-induced inflammation in BV2 cells.

### LPS Activates the TLR4/MyD88 Signaling Pathway in Microglia

**Figure 2:**
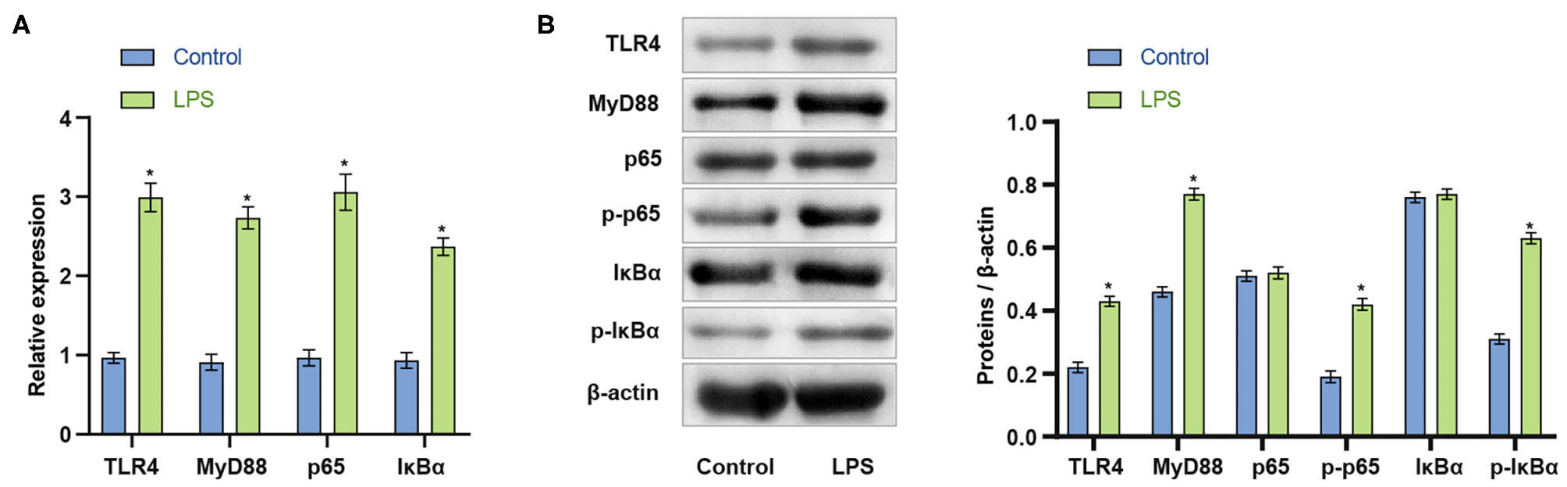
LPS activates the TLR4/MyD88 signaling pathway in microglia. **(A)** The expression of TLR4, MyD88, p65, and IκBα increased after LPS stimulation of BV2 cells. **(B)** Western blot measurement of the expression of MyD88, TLR4, p65, p-p65, IκBα, and p-IκBα protein in BV2 cells. ^*^P < 0.05 compared with the Control group. The unpaired *t*-test was used to analyze comparisons between two groups.

The initial results indicated that LPS could induce apoptosis of BV2 cells. We next explored whether LPS could affect the viability of BV2 cells by activating the TLR4/MyD88 signaling pathway. qRT-PCR was used to detect the expression of TLR4/MyD8. In LPS stimulated BV2 cells, the mRNA expression of TLR4, MyD88, p65, and IκBα increased (*p* < 0.001) (Figure 2A). The findings indicated that LPS might affect the TLR4/MyD88 signaling pathway. LPS stimulation of BV2 also significantly increased the protein expression levels of TLR4, MyD88, p-p65, and p-IκBα (*p* < 0.001), with no significant change in p65 and IκBα (*p* > 0.05) (Figure 2B). The findings indicate that LPS may promote the activation of the TLR4/MyD88 signaling pathway in microglia.

### Inflammatory Response Is Enhanced in SCI Mice, and SCI Is Aggravated

**Figure 3:**
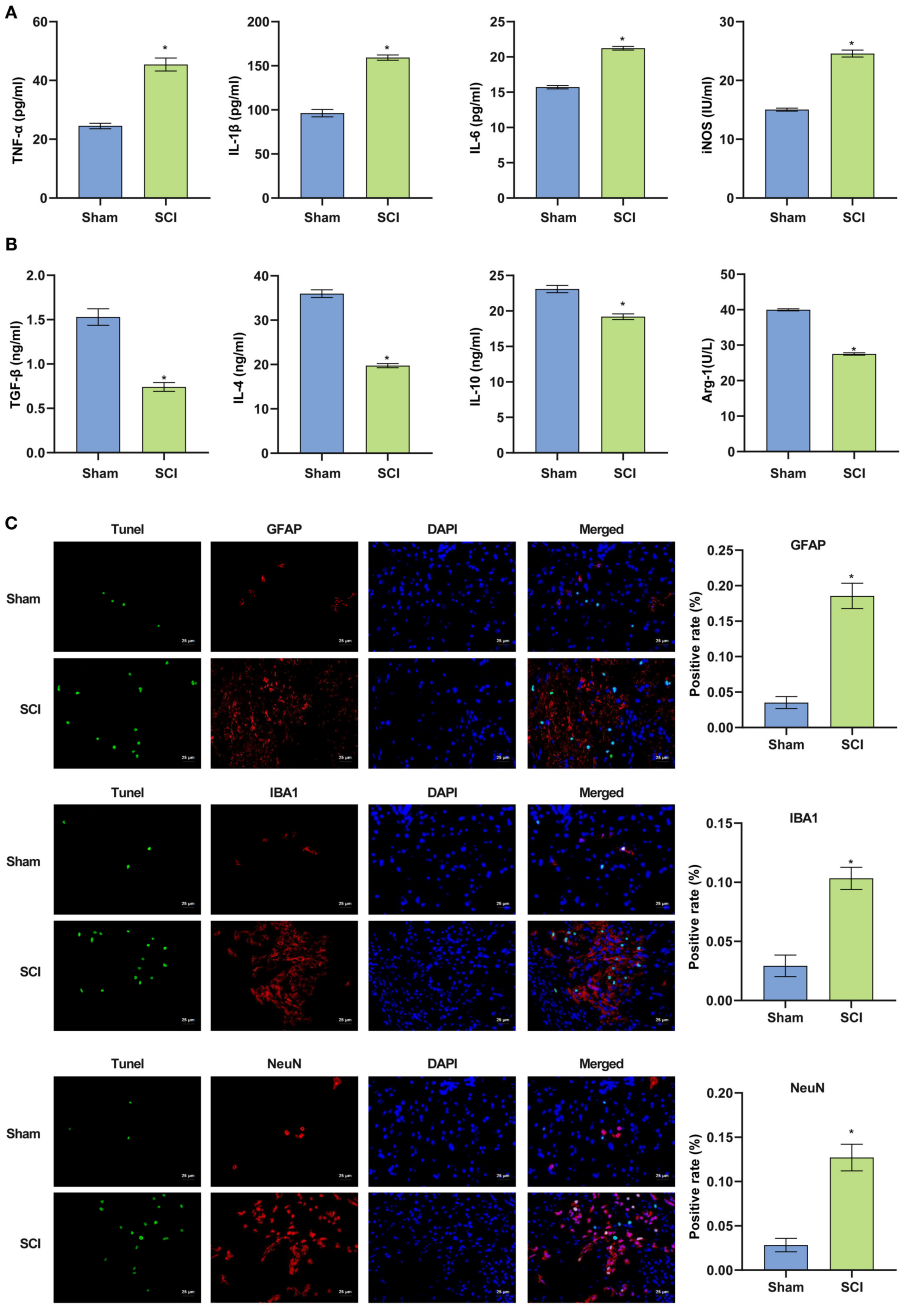
The successful construction of the SCI mouse model. **(A,B)** Pro-inflammatory factors were increased in SCI mice and anti-inflammatory factors were decreased. **(C)** Terminal deoxynucleotidyl transferase dUTP nick end labeling assay was used to detect cell apoptosis in the spinal cord of mice (Scale bar = 25μm). ^*^P < 0.05 compared with Sham group. The unpaired t-test was used to analyze comparisons between two groups *n* = 5.

The above experiments showed that the inflammatory response in the SCI model *in vitro* was enhanced, and that the apoptosis of BV2 cells was intensified. Next, an SCI mouse model was constructed. The results of ELISA experiments in Figures 3A,B demonstrated that compared with the Sham group, serum pro-inflammatory factors (TNF-α, IL-1β, IL-6) and nitric oxide synthase (iNOS) in SCI mice were significantly higher (*p* < 0.001). Increased, anti-inflammatory factors (TGF-β, IL-4, IL-10) and arginase 1 (Arg-1) decreased significantly. The findings indicated the successful construction of the SCI model (*p* < 0.001). The TUNEL and immunofluorescence co-localization experiment was performed on spinal cord sections of Sham and SCI mice to determine the survival and apoptotic cells in the spinal cord. Compared with the Sham group, the fluorescence intensity of the neuron marker (NeuN), microglia marker (IBA1) and astrocyte marker (GFAP) increased in the spinal cord tissue in SCI group. The results suggested that the body may activate neuron, microglia and astrocyte cells to repair the damage when the spinal cord was injured. The amount of apoptotic neurons, microglia and astrocyte also increased, suggesting that spinal cord injury could cause apoptosis at a certain degree (*p* < 0.001) (Figure 3C). In fact, the amounts of both survival and apoptotic neuron, microglia and astrocyte increased based on the fluorescence images from the co-localization experiments of TUNEL and immunofluorescence.

### Activation of the TLR4/MyD88 Signaling Pathway in the Spinal Cord and Colon of SCI Mice

**Figure 4:**
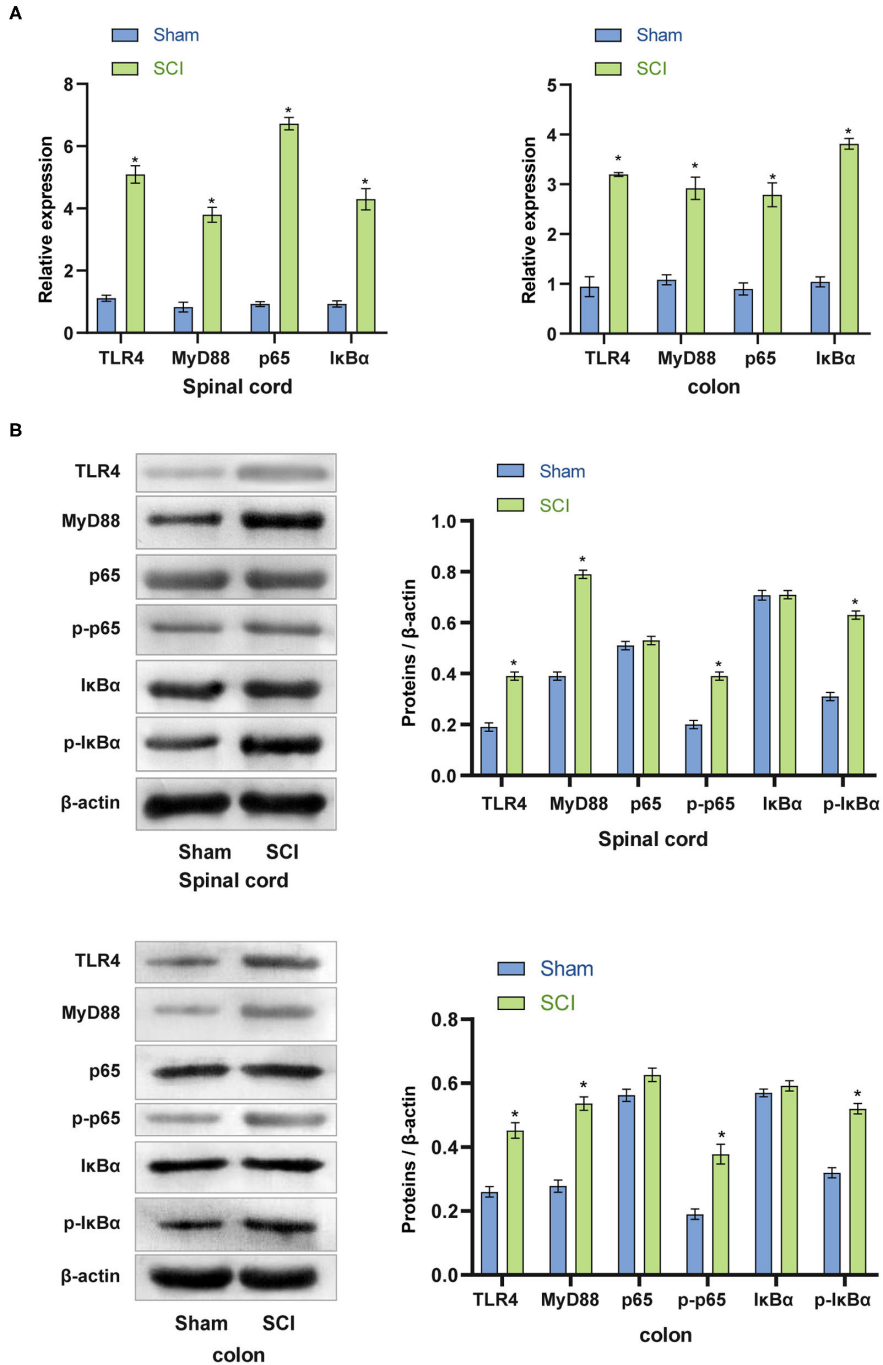
Activation of the TLR4/MyD88 signaling pathway in the spinal cord and colon of SCI mice. **(A)** qRT-PCR was used to analyze the expression of TLR4, MyD88, p65, IκBα in the spinal cord and colon tissues. **(B)** The protein expression of TLR4, MyD88, p-p65, and p-IκBα increased significantly in SCI mice. ^*^P < 0.05 compared with the Sham group. The unpaired *t*-test was used to analyze comparisons between two groups *n* = 5.

To further investigate whether the TLR4/MyD88 signaling pathway was activated *in vivo*, we removed spinal cord and colon tissues from the SCI mice for qRT-PCR and western blot analyses. Compared with the Sham group, the mRNA expression of TLR4, MyD88, p65, and IκBα in the spinal cord and colon tissues of the SCI group was considerably increased (*p* < 0.001) (Figure 4A). The data indicated that the TLR4/MyD88 signaling pathway was activated. Western blot examination was used to detect the TLR4/MyD88 signaling pathway in the spinal cord and colon tissue. The expression of TLR4, MyD88, p-p65, and p-IκBα increased dramatically (*p* < 0.001), while the expression of non-phosphorylated p65 and IκBα did not increase significantly (*p* < 0.001) (Figure 4B). The collective findings indicate that the TLR4/MyD88 signaling pathway was activated in SCI mice.

### Gut Microbiota Imbalance in SCI Mice

**Figure 5:**
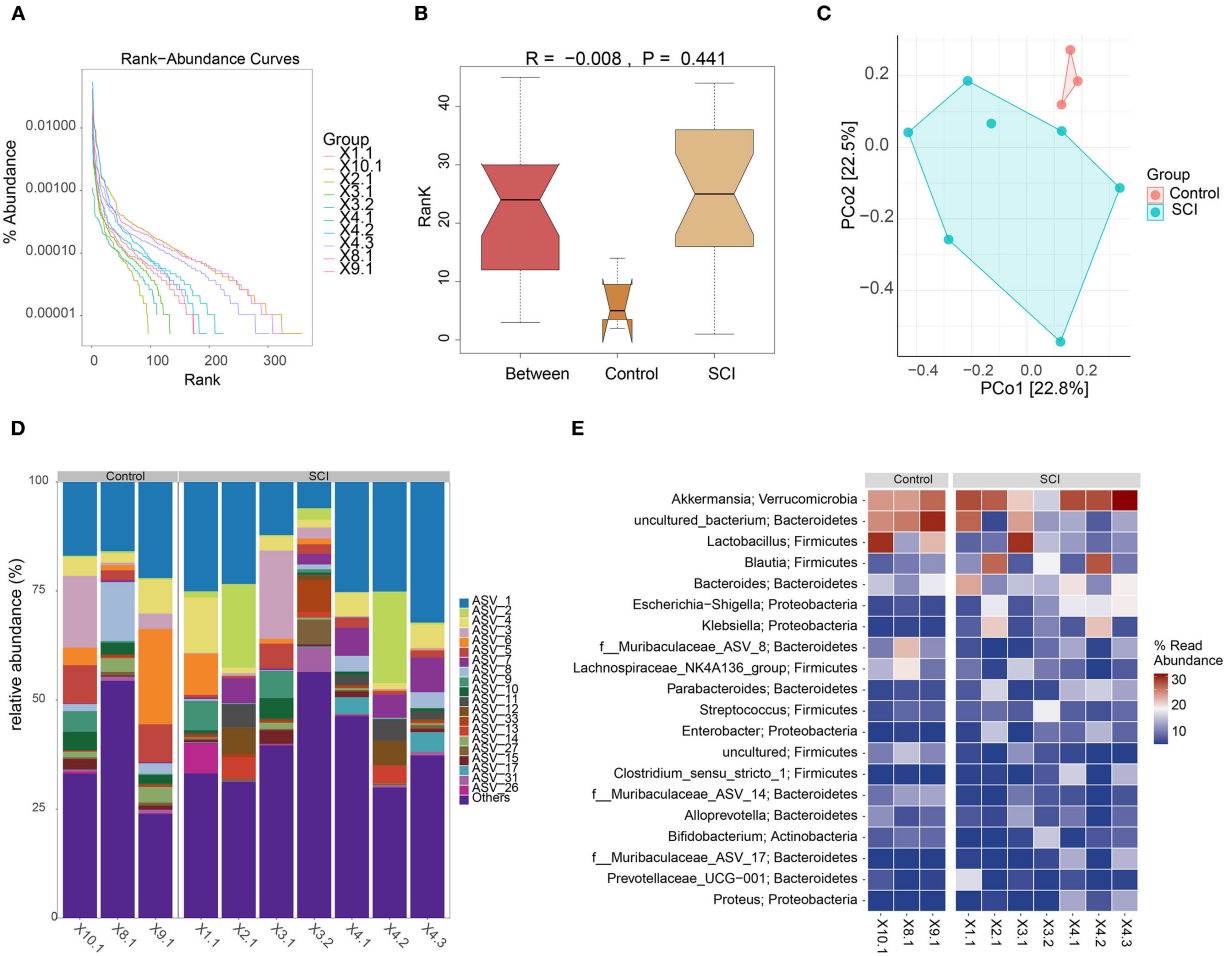
Gut microbiota analyses. **(A)** Rank-abundance curve. **(B)** Box plot of diversity differences between the analyses of similarities groups. **(C)** Principal component analysis plot. **(D)** Histogram of species distribution. **(E)** Operational taxonomic unit abundance clustering heat map.

The above experimental results indicated that the inflammatory response of the SCI model *in vivo* was increased, and the TLR4/MyD88 signaling pathway was activated. We speculated that these physiological phenomena might reflect changes in the gut microbiota of SCI mice. Rank-abundance curve analysis revealed that the SCI group curve had a smaller range on the horizontal axis, indicating that the species abundance was the lowest (Figure 5A). The distance matrix between the samples was analyzed (R = −0.008) (Figure 5B). Although the difference between the Control and SCI groups was not noticeable, the principal component analysis revealed differences in microbial communities in the two samples (Figure 5C). The farther the distance, the lower was the similarity. The species distribution map and operational taxonomic unit (out) abundance clustering heat map indicated a difference in bacterial population distribution between the control and SCI groups (Figures 5D,E). The collective findings were indicative of gut microbiota deregulation in SCI mice.

### Gut Microbiota Imbalance Activates the TLR4/MyD88 Signaling Pathway

**Figure 6:**
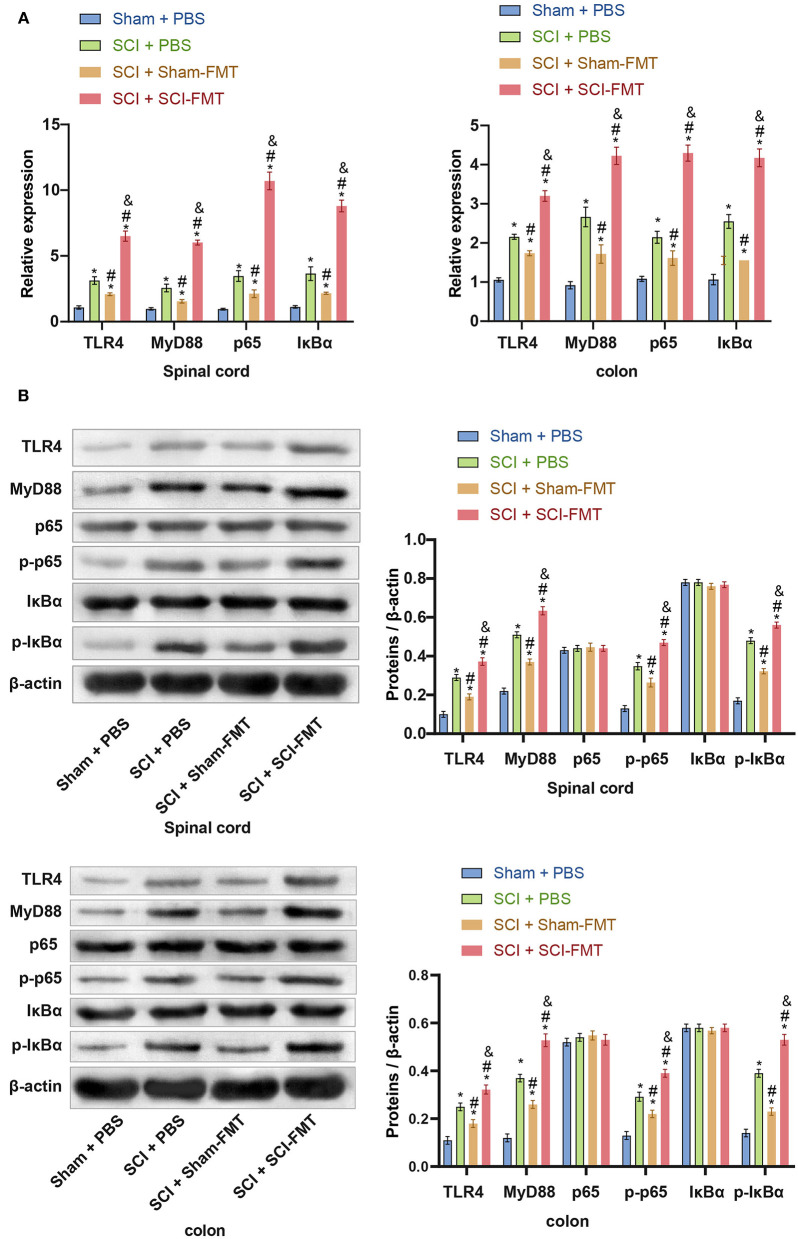
The imbalance of the gut microbiota in SCI mice could activate the TLR4/MyD88 signaling pathway. **(A)** The expression of TLR4, MyD88, p65, and IκBα in the spinal cord and colon tissues. **(B)** Imbalance of the gut microbiota of SCI mice could promote the protein expression of TLR4, MyD88, p-p65, and p-IκBα. ^*^, *P* < 0.05 compared with the Sham + PBS group. #, *P* < 0.05 compared with the SCI + PBS group. &, *P* < 0.05 compared with the SCI + Sham-FMT group. Multiple comparisons in groups were evaluated by one-way analysis of variance *n* = 5.

We speculated that activation of the mouse TLR4/MyD88 signaling pathway is related to an imbalance in the gut microbiota. To assess this, we performed FMT in mice to detect the expression of the TLR4/MyD88 signaling pathway in the spinal cord and colon. Compared with the SCI + Sham-FMT group, the mRNA expression of TLR4, MyD88, p65, and IκBα in the SCI + SCI-FMT group increased sharply and was more significant than that in the SCI + phosphate-buffered saline (PBS) group (*p* < 0.001). The data indicated that the imbalance of gut microbiota could promote the activation of the TLR4/MyD88 signaling pathway (Figure 6A). Next, we used western blot to detect the expression of proteins in the TLR4/MyD88 signaling pathway in spinal cord tissue and colon tissue. Compared with the SCI + Sham-FMT group, the data of the SCI + SCI-FMT group showed that the expression of TLR4, MyD88, p-p65, and p-IκBα increased dramatically (*p* < 0.001), while the levels of non-phosphorylated p65 and IκBα were not significantly different (*p* < 0.001) (Figure 6B). The collective findings supported the view that the gut microbiota imbalance in SCI mice could activate the TLR4/MyD88 signaling pathway.

**Figure 7:**
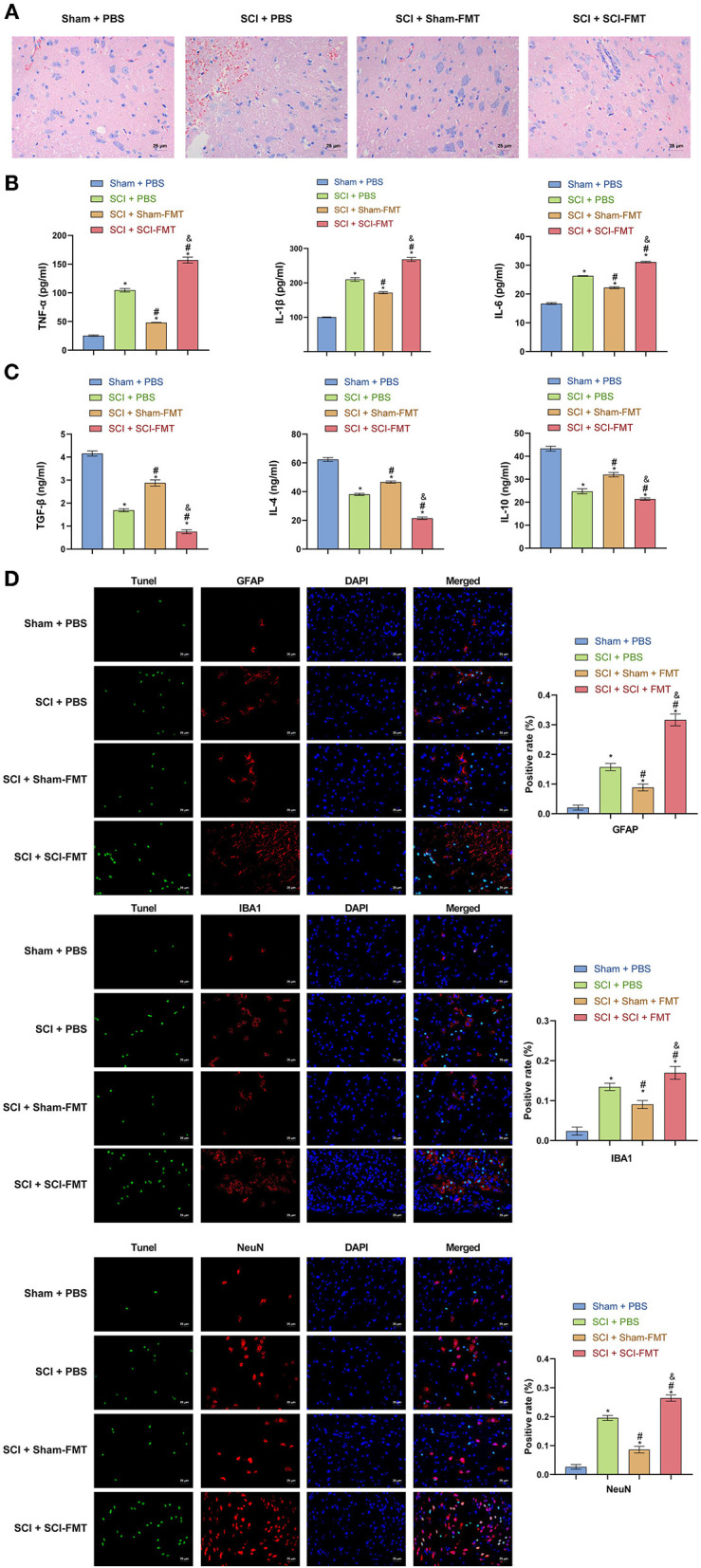
Imbalance of gut microbiota exacerbates SCI. **(A)** Hematoxylin and eosin staining was used to detect SCI in each group. **(B, C)** ELISA was used to observe the content of inflammatory factors in the serum of mice in each group. **(D)** TUNEL assay and immunofluorescence detection of spinal cord cell apoptosis in each group of mice (Scale bar = 25 μm). ^*^, *P* < 0.05 compared with the Sham + PBS group. #, *P* < 0.05 compared with the SCI + PBS group. &, *P* < 0.05 compared with the SCI +Sham-FMT group. Multiple comparisons in groups were evaluated by one-way analysis of variance *n* = 5.

The data in Figure 6 indicated that an imbalance of the gut microbiota could activate the TLR4/MyD88 signaling pathway, promote inflammation, and exacerbate SCI. To study this further, we analyzed explored mice following FMT. Compared to Sham + PBS mice, the visible damage to SCI + PBS mice was more serious. The SCI + SCI + FMT group displayed the most severe SCI (*p* < 0.001) (Figure 7A). The results showed that the imbalance of gut microbiota exacerbated SCI. ELISA determined the levels of pro-inflammatory cytokines and anti-inflammatory cytokines in the serum. The increase in pro-inflammatory cytokines (TNF-α, IL-1β, and IL-6) in the SCI + SCI + FMT group was the most obvious, and the anti-inflammatory cell factors (TGF-β, IL-4, and IL-10) were most severely inhibited (*p* < 0.001) (Figures 7B,C). Finally, the fluorescence intensity of GFAP, NeuN and IBA1 was the highest in SCI + SCI + FMT. It showed that fecal transplantation could increase the amount of survival neuron, microglia and astrocyte cells at the site of spinal cord injury (*p* < 0.001) (Figure 7D). The collective results showed that an imbalance in gut microbiota could promote inflammation and exacerbate SCI.

## Discussion

The gut microbiota of mice with SCI was disordered, which caused systemic inflammation. After FMT in mice with SCI, the injury aggravated systemic inflammation. *In vitro*, LPS activated the TLR4/MyD88 signaling pathway in microglia, inducing the production of inflammatory cytokines and increasing microglial apoptosis. The findings indicate that TLR4/MyD88 pathway signal transduction may be related to the aggravation of SCI caused by the imbalanced gut microbiota.

SCI refers to the direct or indirect external damage to normal spinal and spinal cord tissues, which can affect spinal cord function. Recent data from rodents indicate that SCI causes gut dysbiosis, which exacerbates intraspinal inflammation and lesion pathology leading to impaired recovery of motor function. Postinjury delivery of probiotics containing various types of “good” bacteria can partially overcome the pathophysiologic effects of gut dysbiosis. Immune function, locomotor recovery, and spinal cord integrity are partially restored by a sustained regimen of oral probiotics (5). Firmicutes and Bacteroides spp. are the most predominant phylum in the gut. They ferment non-digestible polysaccharides and generate metabolites that can be used for energy by the host. Acetate, propionate and butyrate are among the most well characterized single chain fatty acid metabolites that are produced following carbohydrate fermentation in the gut. Short chain fatty acids, butyrate in particular, have potent anti-inflammatory effects on macrophages and can suppress ongoing inflammation in the Central nervous system (20). Gut microbiota is a key and potential target for SCI (4, 7), although its crosstalk with the TLR4/MyD88 signaling pathway during SCI is still unclear (21). The gut microbiota relies on the TLR4 pathogen recognition receptor, which interacts with the host. TLR4 recognizes LPS in gram-negative bacteria and activates downstream pro-inflammatory signaling, including the TLR4/MyD88/p38 mitogen-activated protein kinase pathway, which is also thought to be involved in the regulation of TJPs and intestinal permeability (22, 23). Compared with wild-type mice, TLR4-/- mice reportedly show less pancreatic damage and inflammation in pancreatitis, which supports the importance of TLR4 in the pathogenesis of pancreatitis. TLR4 has also been shown to cause intestinal damage. TLR4 signal transduction increased the immunopathology of the ileum in TLR4-/- mice (24). Neuroinflammation plays a crucial role in the secondary phase of SCI, and is initiated following the activation of TLR4. Pyroptosis is a form of inflammatory programmed cell death, which is closely involved in neuroinflammation, and it can be regulated by TLR4 according to a recent research (12). BMSCs-derived exosomes could inhibit apoptosis and inflammation response induced by spinal cord injury and promote motor function recovery by inhibiting the TLR4/MyD88/NF-κB signaling pathway (25). In the present study, FMT led to the activation of the TLR4/MyD88 signaling pathway in the transplanted mice and aggravation of SCI was. The data verified that the gut microbiota may be a key target of SCI and may target TLR4.

Activation of the TLR4/MyD88 pathway and upregulation of the TNF-α, IL-12, and IL-6 inflammatory cytokines are involved in the development of SCI, which are closely related to neuroinflammatory injury and can be used as a reference index to evaluate the prognosis of SCI patients (26). TLR4/MyD88 was shown to activate the nuclear factor-kappa B (NF-κB) inflammatory pathway in enteritis (27), promote myocardial infarction (28) and induce acute pneumonia (29). As a key component of the inflammatory microenvironment, inflammatory factors also play a key role in the repair of nerve damage after SCI. This involves pro-inflammatory factors, including IL-1β, TNF-αIL-6, and β, IL-4, IL-10 and IL-13 (30). Most TLRs perform their functions through the MyD88 pathway (31). GFAP and IBA1, which are the assigned markers for the activated astrocytes and microglia, were significantly upregulated in the treated group. The inhibition of TLR4 further inhibited the expression of its downstream effectors (IBA1) in the microglial cells (32). Studies hypothesized that suppressed MyD88 adaptor protein in the spinal cord could alleviate peripheral nerve injury-induced neuropathic pain. MyD88 adaptor protein involved in the neuropathic pain and may provide potential therapeutic strategies for treatment of neuropathic pain (33). Previous studies revealed that GFAP and IBA1 labeled astrocyte and microglia increased and NeuN labeled neuron decreased in SCI spinal cord injury (34). Our results showed that GFAP, NeuN and IBA1 labeled cells increased. However, the co-localization results of TUNEL showed that the amount of apoptotic neurons also increased. There may exist several reasons. Firstly, apoptotic cells associated with spinal cord injury cannot be avoided. It is possible that rapid self-repair happened in SCI mice. In a previous study, neurons of spinal cord injury also increased at 8 days after SCI modeling (35). Moreover, endogenous neural stem cells and ependymal stem cells could differentiate into neurons after spinal cord injury (36, 37). Lineage tracing experiments have shown that it is solely the ependymal cell population that is capable of generating neurospheres *in vitro*, and this hallmark of NSC potential remains restricted to the ependymal population following SCI (38). This may also be one of the reasons for the increase in neuron. We are very interested in the emergence of these different results. We plan to study the reasons for the increase in NeuN under spinal cord injury in the future. The results of the present study showed that the activation of the TLR4/MyD88 pathway could trigger the overexpression of pro-inflammatory factors, promote cell apoptosis, and further aggravate nerve damage.

In conclusion, LPS-induced microglia have an inflammatory response, TLR4/MyD88 is activated, and the rate of microglial apoptosis increases. *In vivo*, the disturbance of the gut microbiota in mice with SCI confirmed that gut microbiota disorders can aggravate SCI by activating the TLR4/MyD88 signaling pathway in mice and has potential value as a treatment for SCI and other neuroinflammation-related diseases.

## Data Availability Statement

The datasets presented in this study can be found in online repositories. The names of the repository/repositories and accession number(s) can be found below: https://www.ncbi.nlm.nih.gov/, PRJNA726026.

## Ethics Statement

The animal study was reviewed and approved by the Ethics Committee of Huizhou Municipal Central Hospital.

## Author Contributions

ZR, YH, HC, and JW performed the experiment and analyzed the data. ZR, MC, HW, GL, and ZZ performed the experiment. ZR and JW guided the experiment and edited the manuscript. YH, HC, MC, GL, and HW revised the manuscript. All authors read and approved the final manuscript.

## Funding

This work was partially supported by the funds from the Science and Technology Program of Huizhou (2021WC0106362).

## Conflict of Interest

The authors declare that the research was conducted in the absence of any commercial or financial relationships that could be construed as a potential conflict of interest.

## Publisher's Note

All claims expressed in this article are solely those of the authors and do not necessarily represent those of their affiliated organizations, or those of the publisher, the editors and the reviewers. Any product that may be evaluated in this article, or claim that may be made by its manufacturer, is not guaranteed or endorsed by the publisher.

## References

[1] Martirosyan NL Turner GH. Manganese-enhanced MRI Offers Correlation with Severity of Spinal Cord Injury in Experimental Models. Open Neuroimag J. (2016) 10:139–47. 10.2174/1874440001610010139 28144384 PMC5226969

[2] Orr MB Gensel JC. Spinal cord injury scarring and inflammation: therapies targeting glial and inflammatory responses. Neurotherapeutics. (2018) 15:541–53. 10.1007/s13311-018-0631-6 29717413 PMC6095779

[3] Meng XL Hai Y Zhang XN Wang YS Liu XH Ma LL. Hyperbaric oxygen improves functional recovery of rats after spinal cord injury via activating stromal cell-derived factor-1/CXC chemokine receptor 4 axis and promoting brain-derived neurothrophic factor expression. Chin Med J. (2019) 132:699–706. 10.1097/CM9.0000000000000115 30855350 PMC6416102

[4] Kigerl KA Hall JC Wang L Mo X Yu Z Popovich PG. Gut dysbiosis impairs recovery after spinal cord injury. J Exp Med. (2016) 213:2603–20. 10.1084/jem.20151345 27810921 PMC5110012

[5] Kigerl KA Mostacada K Popovich PG. Gut microbiota are disease-modifying factors after traumatic spinal cord injury. Neurotherapeutics. (2018) 15:60–7. 10.1007/s13311-017-0583-2 29101668 PMC5794696

[6] Jing Y Yang D Bai F Zhang C Qin C Li D. Melatonin treatment alleviates spinal cord injury-induced gut dysbiosis in mice. J Neurotrauma. (2019) 36:2646–64. 10.1089/neu.2018.6012 30693824

[7] O'Connor G Jeffrey E Madorma D Marcillo A Abreu MT Deo SK. Investigation of microbiota alterations and intestinal inflammation post-spinal cord injury in rat model. J Neurotrauma. (2018) 35:2159–66. 10.1089/neu.2017.5349 29566601 PMC6119224

[8] Tilg H Zmora N Adolph TE Elinav E. The intestinal microbiota fuelling metabolic inflammation. Nat Rev Immunol. (2020) 20:40–54. 10.1038/s41577-019-0198-4 31388093

[9] Sun MF Zhu YL Zhou ZL Jia XB Xu YD Yang Q. Neuroprotective effects of fecal microbiota transplantation on MPTP-induced Parkinson's disease mice: gut microbiota, glial reaction and TLR4/TNF-α signaling pathway. Brain Behav Immun. (2018) 70:48–60. 10.1016/j.bbi.2018.02.005 29471030

[10] Sun Z Zhao T. Dexmedetomidine attenuates spinal cord ischemia–reperfusion injury through both anti-inflammation and anti-apoptosis mechanisms in rabbits. J Transl Med. (2018) 16:1–11. 10.1186/s12967-018-1583-7 30031397 PMC6054716

[11] Alhasson F Das S Seth R Dattaroy D Chandrashekaran V Ryan CN. Altered gut microbiome in a mouse model of Gulf War Illness causes neuroinflammation and intestinal injury via leaky gut and TLR4 activation. PLoS ONE. (2017) 12:e0172914. 10.1371/journal.pone.0172914 28328972 PMC5362211

[12] Xu S Wang J Jiang J Song J Zhu W Zhang F. TLR4 promotes microglial pyroptosis via lncRNA-F630028O10Rik by activating PI3K/AKT pathway after spinal cord injury. Cell Death Dis. (2020) 11:693. 10.1038/s41419-020-02824-z 32826878 PMC7443136

[13] Mi S Wu Y Hong Z Wang Z Feng X Zheng G. Expression of TLR4/MyD88/NF-κB pathway genes and its related inflammatory factors in secondary spinal cord injury. Zhejiang Da Xue Xue Bao Yi Xue Ban. (2019) 48:609–16. 31955534 10.3785/j.issn.1008-9292.2019.12.04 PMC8800780

[14] Ribeiro P Castro MV Perez M Cartarozzi LP Spejo AB Chiarotto GB. Toll-like receptor 4 (TLR4) influences the glial reaction in the spinal cord and the neural response to injury following peripheral nerve crush. Brain Res Bull. (2020) 155:67–80. 10.1016/j.brainresbull.2019.11.008 31756421

[15] Xu J Lu C Liu Z Zhang P Guo H Wang T. Schizandrin B protects LPS-induced sepsis via TLR4/NF-κB/MyD88 signaling pathway. Am J Transl Res. (2018) 10:1155–63. 29736208 PMC5934574

[16] Ryu J-K Kim SJ. Reconstruction of LPS transfer cascade reveals structural determinants within LBP, CD14, and TLR4-MD2 for efficient LPS recognition and transfer. Immunity. (2017) 46:38–50. 10.1016/j.immuni.2016.11.007 27986454

[17] Yu F-Z Wen X. L6H21 prolonged rats survival after limb allotransplantation by inhibiting acute rejection. Eur Rev Med Pharmacol Sci. (2017) 21:1891–903. 28485788

[18] Saikh KU. MyD88 and beyond: a perspective on MyD88-targeted therapeutic approach for modulation of host immunity. Immunol Res. (2021) 69:117–28. 10.1007/s12026-021-09188-2 33834387 PMC8031343

[19] Hu N Wang C. Phillygenin inhibits LPS-induced activation and inflammation of LX2 cells by TLR4/MyD88/NF-κB signaling pathway. J Ethnopharmacol. (2020) 248:112361. 10.1016/j.jep.2019.112361 31683033

[20] Gungor B Adiguzel E. Intestinal microbiota in patients with spinal cord injury. PLoS ONE. (2016) 11:e0145878. 10.1371/journal.pone.0145878 26752409 PMC4709077

[21] Zheng J Lou L. Commensal escherichia coli aggravates acute necrotizing pancreatitis through targeting of intestinal epithelial cells. Appl Environ Microbiol. (2019) 85:e00059–19. 10.1128/AEM.00059-19 30979838 PMC6544826

[22] Ouyang J. Up-regulation of tight-junction proteins by p38 mitogen-activated protein kinase/p53 inhibition leads to a reduction of injury to the intestinal mucosal barrier in severe acute pancreatitis. Pancreas. (2016) 45:1136–44. 10.1097/MPA.0000000000000656 27171513

[23] Liu L Jiang Y. Toll-like receptor 4 regulates occludin and zonula occludens 1 to reduce retinal permeability both *in vitro* and *in vivo*. J Vasc Res. (2018) 54:367–75. 10.1159/000480455 29136627 PMC5896294

[24] Kløve S Genger C. Toll-like receptor-4 is involved in mediating intestinal and extra-intestinal inflammation in campylobacter coli-infected secondary abiotic il-10 -/- mice. Microorganisms. (2020) 8:1882. 10.3390/microorganisms8121882 33261211 PMC7761268

[25] Fan L Dong J. Bone marrow mesenchymal stem cells-derived exosomes reduce apoptosis and inflammatory response during spinal cord injury by inhibiting the TLR4/MyD88/NF-κB signaling pathway. Hum Exp Toxicol. (2021) 29:09603271211003311. 10.1177/09603271211003311 33779331

[26] Mi S Wu Y. Expression of TLR4/MyD88/NF-κB pathway genes and its related inflammatory factors in secondary spinal cord injury. Zhejiang Da Xue Xue Bao Yi Xue Ban. (2019) 48:609–16. 31955534 10.3785/j.issn.1008-9292.2019.12.04 PMC8800780

[27] Zhou M Xu W. Boosting mTOR-dependent autophagy via upstream TLR4-MyD88-MAPK signalling and downstream NF-κB pathway quenches intestinal inflammation and oxidative stress injury. EBioMedicine. (2018) 35:345–60. 10.1016/j.ebiom.2018.08.035 30170968 PMC6161481

[28] Xu G-R Zhang C. Modified citrus pectin ameliorates myocardial fibrosis and inflammation via suppressing galectin-3 and TLR4/MyD88/NF-κB signaling pathway. Biomed Pharmacother. (2020) 126:110071. 10.1016/j.biopha.2020.110071 32172066

[29] Liu G Lu Y. TLR4-MyD88 signaling pathway is responsible for acute lung inflammation induced by reclaimed water. J Hazard Mater. (2020) 396:122586. 10.1016/j.jhazmat.2020.122586 32315938

[30] Bank M Stein A. Elevated circulating levels of the pro-inflammatory cytokine macrophage migration inhibitory factor in individuals with acute spinal cord injury. Arch Phys Med Rehabil. (2015) 96:633–44. 10.1016/j.apmr.2014.10.021 25461821

[31] Zhu X Liu J. Neuroprotective and anti-inflammatory effects of isoliquiritigenin in kainic acid-induced epileptic rats via the TLR4/MYD88 signaling pathway. Inflammopharmacology. (2019) 27:1143–53. 10.1007/s10787-019-00592-7 31037573

[32] Ikram M Saeed K. Natural dietary supplementation of curcumin protects mice brains against ethanol-induced oxidative stress-mediated neurodegeneration and memory impairment via Nrf2/TLR4/RAGE signaling. Nutrients. (2019) 11:1082. 10.3390/nu11051082 31096703 PMC6566393

[33] Liu F Wang Z. Suppression of MyD88-dependent signaling alleviates neuropathic pain induced by peripheral nerve injury in the rat. J Neuroinflammation. (2017) 14:1–12. 10.1186/s12974-017-0822-9 28359290 PMC5374701

[34] Qian J Zhu W Lu M Ni B Yang J. D-β-hydroxybutyrate promotes functional recovery and relieves pain hypersensitivity in mice with spinal cord injury. Br J Pharmacol. (2017) 174:1961–71. 10.1111/bph.13788 28320049 PMC5466532

[35] Torres BB Caldeira FM Gomes MG Serakides R de Marco Viott A Bertagnolli AC. Effects of dantrolene on apoptosis and immunohistochemical expression of NeuN in the spinal cord after traumatic injury in rats. Int J Exp Pathol. (2010) 91:530–6. 10.1111/j.1365-2613.2010.00738.x 21039984 PMC3010552

[36] Zhang L Fan C Hao W Zhuang Y Liu X Zhao Y. NSCs migration promoted and drug delivered exosomes-collagen scaffold via a bio-specific peptide for one-step spinal cord injury repair. Adv Healthc Mater. (2021) 10:e2001896. 10.1002/adhm.202001896 33522126

[37] Grégoire CA Goldenstein BL Floriddia EM Barnabé-Heider F Fernandes KJ. Endogenous neural stem cell responses to stroke and spinal cord injury. Glia. (2015) 63:1469–82. 10.1002/glia.22851 25921491

[38] Barnabé-Heider F Göritz C Sabelström H Takebayashi H Pfrieger FW Meletis K. Origin of new glial cells in intact and injured adult spinal cord. Cell Stem Cell. (2010) 7:470–82. 10.1016/j.stem.2010.07.014 20887953

